# A new species of *Cosmocerca* (Nematoda, Ascaridomorpha) from the marine toad *Rhinella marina* (Linnaeus) (Anura, Bufonidae) in Australia

**DOI:** 10.3897/zookeys.931.50478

**Published:** 2020-04-30

**Authors:** Xue-Feng Ni, Diane P. Barton, Hui-Xia Chen, Liang Li

**Affiliations:** 1 Key Laboratory of Animal Physiology, Biochemistry and Molecular Biology of Hebei Province, College of Life Sciences, Hebei Normal University, 050024 Shijiazhuang, Hebei Province, China Hebei Normal University Shijiazhuang China; 2 School of Tropical Biology, James Cook University, Townsville, Queensland 4811, Australia James Cook University Townsville Australia; 3 School of Animal and Veterinary Sciences, Charles Sturt University, Estella, New South Wales 2678, Australia Charles Sturt University Estella Australia

**Keywords:** parasite, nematode, Ascaridida, Cosmocercoidea, marine toad *Rhinella marina*, new species, Australasian Region

## Abstract

The marine toad *Rhinella marina* (Linnaeus) (Anura, Bufonidae) is a notorious, exotic amphibian species in Australia. However, our present knowledge of the composition of the nematode fauna of *R. marina* is still not complete. In the present study, a new cosmocercid nematode, *Cosmocerca multipapillata***sp. nov.**, was described using both light and scanning electron microscopy, based on specimens collected from *R. marina* in Australia. *Cosmocerca multipapillata***sp. nov.** can be easily distinguished from its congeners by the body size, the presence of lateral alae and well sclerotized gubernaculum, the number and arrangement of plectanes and rosettes and the length of spicules, oesophagus and tail.

## Introduction

The marine toad *Rhinella marina* (Linnaeus) (Anura, Bufonidae) is a large, terrestrial toad, which is natively distributed in Central and South America (Zug and Zug 1979; Lever 2001). The species has been widely introduced to the United States, Fiji, Philippines, Papua New Guinea, Australia, Japan, the Caribbean and some Pacific islands for controlling agricultural pests (Alford et al. 1995; Frost 2016). The helminth fauna of *R. marina* was studied by many authors and over 30 species of nematode parasites have been recorded from this host (Brenes and Bravo-Hollis 1959; Speare 1990; Goldberg and Bursey 1992; Barton 1996; Bursey et al. 2000; Kuzmin et al. 2007; Espinoza-Jimenez et al. 2007; Bursey and Brooks 2010; Drake et al. 2014).

During a helminthological survey in Australian amphibians, some nematodes belonging to the Cosmocercoidea Travassos, 1925 were collected from *R. marina*. Their examination using light and scanning electron microscopy revealed that these specimens represented a new species of *Cosmocerca* Diesing, 1861.

## Materials and methods

### Light and scanning electron microscopy

Nematodes were collected from the intestine of the marine toad *R. marina* (Linnaeus) (Anura, Bufonidae) in various locations from Queensland, Australia. Specimens were fixed and stored in 70% ethanol until study. For light microscopy studies, nematodes were cleared in lactophenol. Drawings were made with the use of a Nikon microscope drawing attachment. For scanning electron microscopy (SEM), specimens were re-fixed in a 4% formaldehyde solution, post-fixed in 1% OsO4, dehydrated via an ethanol series and acetone, and then critical point dried. Samples were coated with gold and examined using a Hitachi S-4800 scanning electron microscope at an accelerating voltage of 20 kV. Measurements (the range, followed by the mean in parentheses) are given in micrometers (μm) unless otherwise stated. Type specimens were deposited in College of Life Sciences, Hebei Normal University, Hebei Province, China.

## Results

### Cosmocerca multipapillata sp. nov.

Taxon classificationAnimaliaRhabditidaCosmocercidae

86B87095-681D-5786-9760-010767B5CB70

http://zoobank.org/45496476-7E22-4A91-A0F5-E5CFFA7E9123

Figures 1 , 2 , 3

#### Description.

**Figure 1.**
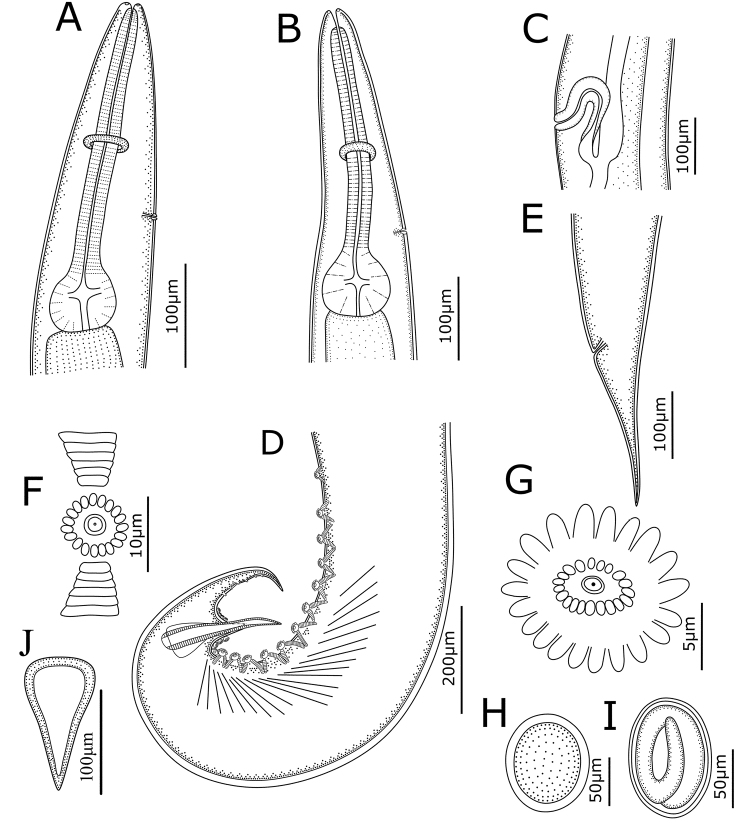
*Cosmocerca multipapillata* sp. nov. collected from the marine toad *Rhinella marina* (Linnaeus) (Anura: Bufonidae) in Australia. **A** anterior part of male, lateral view **B** anterior part of female, lateral view **C** region of vulva, lateral view **D** posterior end of male, lateral view **E** posterior end of female, lateral view **F, G** plectane **H, I** eggs **J** gubernaculum.

**Figure 2.**
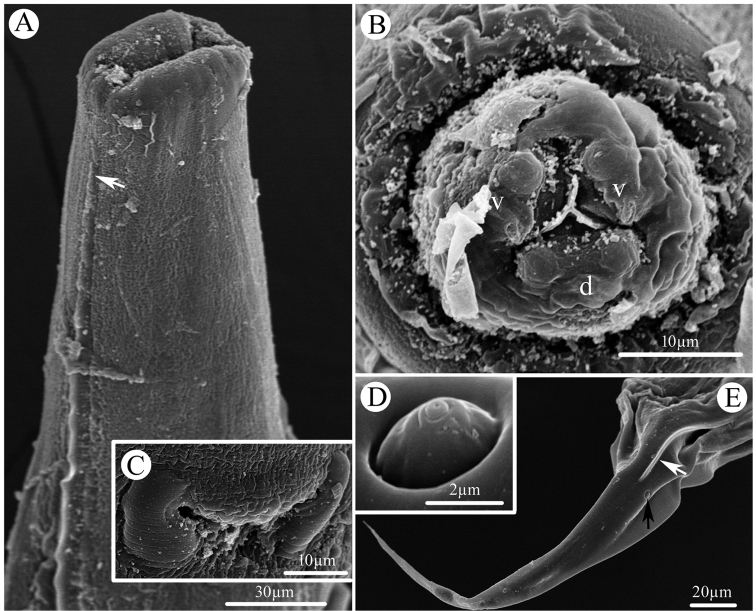
Scanning electron micrographs of female *Cosmocerca multipapillata* sp. nov collected from the marine toad *Rhinella marina* (Linnaeus) (Anura: Bufonidae) in Australia. **A** anterior part of body (lateral ala arrowed), ventrolateral view **B** cephalic end, apical view **C** magnified image of excretory pore **D** magnified image of somatic papilla **E** tail (lateral ala indicated by white arrow, somatic papilla indicated by black arrow), lateral view. Abbreviations: d, dorsal lip; v, ventrolateral lip.

**Figure 3.**
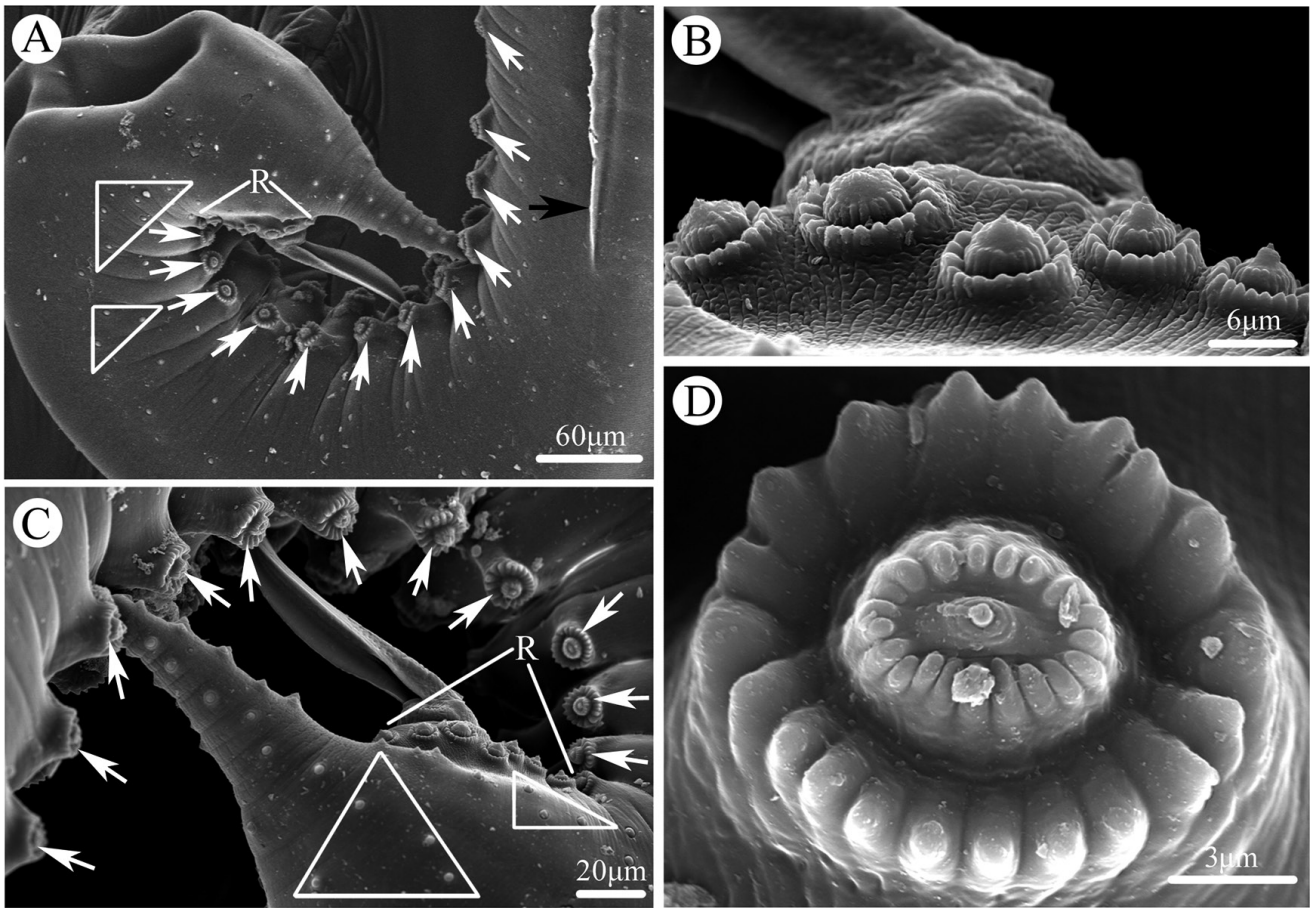
Scanning electron micrographs of male *Cosmocerca multipapillata* sp. nov. collected from the marine toad *Rhinella marina* (Linnaeus) (Anura: Bufonidae) in Australia. **A** posterior end of body (lateral ala indicated by black arrow, plectanes indicated by white arrows, somatic papillae indicated by triangle), sublateral view **B** magnified image of paracloacal rosettes **C** tail (plectanes indicated by white arrows, somatic papillae indicated by triangle), sub-dorsal view **D** magnified image of plectane. Abbreviation: R, rosettes.

Small-sized, whitish nematodes. Body cylindrical, maximum width at about region of mid-body. Cuticle with fine transverse striations. Excretory pore situated slightly anterior to esophageal bulb (Figs 1A, B, 2C). Deirids not observed. Somatic papillae present (Figs 2D, E, 3A, C). Oral aperture simple, somewhat triangular, surrounded by 3 small lips (Fig. 2B). Dorsal lip with one pair of large double cephalic papillae, subventral lips with single large double cephalic papilla and amphid (Fig. 2B). Oesophagus divided into anterior indistinct pharynx, cylindrical corpus and terminal posterior bulb with valves (Fig. 1A, B). Nerve ring located at about 1/2 of oesophageal length. Tail of both sexes conical, with pointed tip (Figs 1D, E, 2E).

**Male** (based on 3 mature specimens): Body 3.10‒3.55 (3.36) mm long; maximum width 248‒327 (297). Oesophagus 365‒479 (406) long (including bulb), representing 10.6‒15.5 (12.2) % of body length; pharynx and corpus 288‒385 (328) long, size of bulb 65‒94 (78.5) × 73‒100 (83.3). Nerve ring 160‒215 (196) and excretory pore 260‒417 (323) from anterior extremity, respectively. Lateral alae narrow, extending from slightly posterior to cephalic end to level of third precloacal plectane (Fig. 2A). Posterior end of body distinctly ventrally curved (Figs 1D, 3A). Spicules alate, equal in length, 169‒219 (185) long, distal end pointed (Figs 1D, 3C), representing 4.75‒6.93 (5.53) % of body length. Gubernaculum small, well sclerotized, 125‒146 (135) long (Fig. 1J). A total of 10–12 pairs of subventral precloacal plectanes and 3‒4 pairs of precloacal rosettes present (Figs 1D, 3A). Each plectane consisting of a central papilla with two complete circles of 18–21 cuticular tubercles seated on underlying support of sclerotized segments (Figs 1F, G, 3D). Usually 3 pairs of subventral paracloacal and 1–2 pairs of postcloacal rosettes present (Figs 1D, 3A–C). Some pairs of small, sub-ventral and simple papillae (indistinguishable from somatic papillae) located at surface of postcloacal region (Fig. 3C). Tail 187‒208 (201) long, representing 5.28–6.72 (6.03) % of body length (Fig. 1D).

**Female** (based on 10 mature specimens): Body 2.68‒3.73 (3.23) mm long; maximum width 188‒277 (232). Oesophagus 338‒428 (376) mm long (including bulb), representing 9.08‒12.8 (11.7) % of body length; pharynx and corpus 273‒343 (194) long, size of bulb 56‒94 (81.3) ×85‒108 (97.3). Nerve ring 145‒183 (164) and excretory pore 259‒329 (281) from anterior extremity, respectively. Lateral alae extending from slightly posterior to cephalic end to level of about 1/2 length of tail. Vulval opening a transverse slit, vulval lips not protruded, 1.24‒1.67 (1.45) mm from anterior extremity, at 41.6‒53.4 (45.5) % of body length (Fig. 1C). Eggs oval, thin-walled with smooth surface, 66‒108 (82.1) × 52‒71 (61) (n = 20) (Fig. 1H, I). Tail 216‒376 (292) long, representing 6.65‒12.4 (9.42) % of body length (Fig. 1E).

### Taxonomic summary

**Type host.** Marine toad *Rhinella marina* (Linnaeus) (Anura, Bufonidae).

**Type locality.** Bloomfield (approximately 180 km north of Cairns), northern Queensland, Australia.

**Other localities.** Cape Tribulation, Port Douglas, Abergowrie, Townsville region, all in northern Queensland, Australia.

**Site of infection.** Rectum.

**Level of infection.** 3.7% (24 out of 643) of *Rhinella marina* specimens were infected, with an intensity of 1–58 (mean 5.2) nematodes.

**Type deposition.** Holotype, male (HBNU–N-2019A024L); allotype, female (HBNU–N-2019A025L); paratypes: 2 males, 120 females (HBNU–N-2019A026L).

**Etymology.** The specific epithet is derived from a combination of the Latin words *multi*- (multiple) and *papillata* (bearing papillae), referring to the characteristic numerous pre-cloacal plectanes.

## Discussion

Species of *Cosmocerca* (Ascaridida, Cosmocercoidea) mainly parasitize the digestive tract of various amphibians (Baker and Green 1988; Moravec and Baruš 1990; Moravec and Kaiser 1994; Rizvi et al. 2011; Sou and Nandi 2015; Sou et al. 2018). Bursey et al. (2015) listed 29 nominal species in this genus. Later, Sou et al. (2018) described a new species, *C. bengalensis* Sou, Sow & Nandi, 2018 from India. To date, a total of 30 species of *Cosmocerca* have been reported worldwide. Among these species, only three have been recorded in the Australasian Region, namely *C. archeyi* Baker & Green, 1988 and *C. australis* Baker & Green, 1988, both from *Leiopelma hochstetteri* Fitzinger (Anura, Leiopelmatidae) in New Zealand, and *C. limnodynastes* Johnson & Simpson, 1942 from *Limnodynastes dorsalis* (Gray) (Anura, Myobatrachidae) in Australia (Johnson and Simpson 1942; Baker and Green 1988; Bursey et al. 2015).

*Cosmocerca multipapillata* sp. nov. can be easily distinguished from the three above-mentioned species by having males with many more plectanes (10‒12 pairs in the new species *vs* only 4‒5 pairs in the other three) and a distinctly longer tail in females (0.22‒0.38 mm, representing 6.65‒12.4% of body length in the new species *vs* 0.14‒0.22 mm, representing 3.25‒6.33% of body length in the other three species) (Johnson and Simpson 1942; Baker and Green 1988; Bursey et al. 2015). Johnson and Simpson (1942) described *C. australiensis* Johnson & Simpson, 1942 and *C. propinqua* Johnson & Simpson, 1942 both from *L. dorsalis* in Australia. Both of them should be treated as *incertae sedis*, because only female specimens were found. In fact, Inglis (1968) considered that *C. australiensis* and *C. propinqua* should be transferred to *Parathelandros* Baylis, 1930 (Oxyurida, Pharyngodonidae) based on the morphological characters of the female. Nevertheless, the new species differs from *C. australiensis* and *C. propinqua* by the distinctly smaller body size in the female (2.68‒3.73 mm in C. multipapillata sp. nov. *vs* 5.0‒9.0 mm in the other two species). In addition, the position of the vulva and the morphology of the female tail of *C. multipapillata* sp. nov. are also different from *C. propinqua* (vulva situated in front of oesophageal bulb in this species). Moreover, the new species has a relatively longer oesophagus than that of *C. australiensis* (oesophageal length representing 9.08‒12.8% of body length in C. multipapillata*vs* representing 3.89‒4.67% of body length in *C. australiensis*) (Baker and Green 1988).

In the genus *Cosmocerca*, *C. ishaqi* (Islam, Farooq & Khanum, 1979) and *C. brasiliensis* Travassos, 1925 have 9 or more pairs of plectanes in males (Islam et al. 1979; Rizvi et al. 2011). *Cosmocerca multipapillata* sp. nov. is different from *C. ishaqi* by having a well sclerotized gubernaculum (0.13‒0.15 mm long), the presence of lateral alae (*vs* gubernaculum and lateral alae absent in *C. ishaqi*) and relatively longer spicules (spicules 0.17‒0.22 mm long, representing 4.75‒6.93% of body length *vs* spicules 0.10 mm long, representing 3.42% of body length) (Islam et al. 1979). *Cosmocerca brasiliensis* was described from *Rhinella crucifer* (Wied) (Anura, Bufonidae), *Ischnocnema guentheri* (Steindachner) (Anura, Brachycephalidae), *Thoropa miliaris* (Spix) (Anura, Cycloramphidae) and *Boana faber* (Wied) (Anura, Hylidae) in Brazil (Travassos 1925, 1931). Dyer and Altig (1976) also reported this parasite in several species of frogs in Ecuador. *Cosmocerca brasiliensis* can be easily distinguished from the new species by having a much larger body size in females (9.0‒12.7 mm long in the former *vs* 2.68‒3.73 mm long in the latter), the absence of paracloacal rosettes and lateral alae (*vs* usually 3 pairs and presence of lateral alae in the new species), and a distinctly longer tail in females (0.53‒0.74 mm long in C. brasiliensis*vs* 0.22‒0.38 mm long in *C. multipapillata* sp. nov.).

Although some previous studies reported the marine toad *R. marina* harboring nematodes belonging to *Cosmocerca* (Speare 1990; Barton 1997; Espinoza-Jimenez et al. 2007), most of these studies did not identify the parasites to species level. Prior to this study, only *C. commutata* (Diesing, 1851), *C. podicipinus* Baker & Vicente, 1984, *C. brasiliensis* and *C. parva* Travassos, 1925 had been recorded in *R. marina* (Skrjabin et al. 1961; Bursey et al. 2001; Bursey and Brooks 2010). However, *C. commutata*, *C. parva* and *C. podicipinus* have only 4–7 pairs of precloacal plectanes (Skrjabin et al. 1961; Baker and Vicente 1984), which easily differentiates them from *C. multipapillata* sp. nov. (presence of 10–12 pairs of precloacal plectanes). The morphological differences between *C. brasiliensis* and *C. multipapillata* sp. nov. have been mentioned previously.

Based on morphological characters of the new species (i.e., the body size, the number of plectanes and the presence of well developed spicules and gubernaculum), we speculate that *C. multipapillata* sp. nov. could have been introduced to Australia along with its host *R. marina*, because all the recorded *Cosmocerca* species in the Australasian Region, including *C. archeyi*, *C. australis* and *C. limnodynastes*, have small body size (body length not over 2.00 mm in males), few plectanes (not over 5 pairs) and rudimental spicules and gubernaculum (Johnson and Simpson 1942; Baker and Green 1988; Bursey et al. 2015). However, some species of *Cosmocerca* found in the Neotropical Region have some common characters with the new species, for example, the body length of *C. brasiliensis*, *C. travasssosi* Rodrigues & Fabio, 1970, *C. cruzi* Rodrigues & Fabio, 1970 and *C. podicipinus* is more or less 3.0 mm or over 3.0 mm in males; *C. brasiliensis*, *C. uruguayensis* Lent & Freitas, 1948 and *C. vrcibradici* Bursey & Goldberg, 2004 all with many plectanes (7–9 pairs); *C. brasiliensis*, *C. rara* Freitas & Vicente, 1966 and *C. vrcibradici* with well developed spicules and/or gubernaculum (Lent and Freitas 1948; Freitas and Vicente 1966; Rodrigues and Fabio 1970; Bursey and Goldberg 2004). However, we need some more direct evidence to elucidate the origin of *C. multipapillata* sp. nov. in the Australasian Region or the Neotropical Region (i.e. if this new species is distributed in the Neotropical Region). Moreover, further studies on the composition of the *Cosmocerca* nematode fauna of native Australasian amphibians and rigorous phylogenetic studies to determine the interspecific relationships of *Cosmocerca* using genetic data including broad representatives worldwide (especially species from the Australasian and Neotropical Regions) are required to solve the evolutionary problem.

## Supplementary Material

XML Treatment for Cosmocerca multipapillata
